# Study protocol: a randomised controlled trial of multiple and single dose activated charcoal for acute self-poisoning

**DOI:** 10.1186/1471-227X-7-2

**Published:** 2007-05-11

**Authors:** Michael Eddleston, Edmund Juszczak, Nick A Buckley, Lalith Senarathna, Fahim Mohammed, Stuart Allen, Wasantha Dissanayake, Ariyasena Hittarage, Shifa Azher, K Jeganathan, Shaluka Jayamanne, MH Rezvi Sheriff, David A Warrell, the Ox-Col Poisoning Study collaborators

**Affiliations:** 1 Centre for Tropical Medicine, Nuffield Department of Clinical Medicine, University of Oxford, UK; 2 Ox-Col Collaboration, Department of Clinical Medicine, Faculty of Medicine, University of Colombo, Sri Lanka; 3 South Asian Clinical Toxicology Research Collaboration, Department of Clinical Medicine, University of Peradeniya, Sri Lanka; 4 Centre for Statistics in Medicine, Wolfson College, University of Oxford, UK; 5 Department of Clinical Pharmacology and Toxicology, Australian National University Medical School, ACT, Australia; 6 Department of Clinical Toxicology, Newcastle Mater Hospital, Newcastle, Australia; 7 Anuradhapura General Hospital, North Central Province, Sri Lanka; 8 Polonnaruwa General Hospital, North Central Province, Sri Lanka

## Abstract

**Background:**

The case fatality for intentional self-poisoning in rural Asia is 10–30 times higher than in the West, mostly due to the use of highly toxic poisons. Activated charcoal is a widely available intervention that may – if given early – bind to poisons in the stomach and prevent their absorption. Current guidelines recommend giving a single dose of charcoal (SDAC) if patients arrive within an hour of ingestion. Multiple doses (MDAC) may increase poison elimination at a later time by interrupting any enterohepatic or enterovascular circulations. The effectiveness of SDAC or MDAC is unknown. Since most patients present to hospital after one hour, we considered MDAC to have a higher likelihood of clinical benefit and set up a study to compare MDAC with no charcoal. A third arm of SDAC was added to help determine whether any benefit noted from MDAC resulted from the first dose or all doses.

**Methods/design:**

We set up a randomised controlled trial assessing the effectiveness of superactivated charcoal in unselected adult self-poisoning patients admitted to the adult medical wards of three Sri Lankan secondary hospitals. Patients were randomised to standard treatment or standard treatment plus either a single 50 g dose of superactivated charcoal dissolved in 300 ml of water or six doses every four hours. All patients with a history of poison ingestion were approached concerning the study and written informed consent taken from each patient, or their relative (for unconscious patients or those <16 yrs), recruited to the study. The exclusion criteria were: age under 14 yrs; prior treatment with activated charcoal during this poisoning episode; pregnancy; ingestion of a corrosive or hydrocarbon; requirement for oral medication; inability of the medical staff to intubate the patient with a Glasgow Coma Score <13; presentation >72 hrs post-ingestion, and previous recruitment. The primary outcome was in-hospital mortality; secondary outcomes included the occurrence of serious complications (need for intubation, time requiring assisted ventilation, fits, cardiac dysrhythmias). Analysis will be on an intention-to-treat basis; the effects of reported time to treatment after poisoning and status on admission will also be assessed.

**Discussion:**

This trial will provide important information on the effectiveness of both single and multiple dose activated charcoal in the forms of poisoning commonly seen in rural Asia. If charcoal is found to be effective, it should be possible to make it widely available across rural Asia in an affordable formulation.

**Trial registration:**

Current Controlled Trials ISRCTN02920054

## Background

Deliberate self-poisoning is a major clinical problem in many parts of the developing world where highly toxic poisons and sparse medical facilities ensure a high fatality rate [1,2]. Pesticides are the major problem – the WHO estimates that they cause more than 220,000 deaths globally each year, of which most are due to organophosphorus (OP) insecticides [3]. But other poisons, in particular plants, other pesticides, and some pharmaceuticals are locally important problems [1].

Self-poisoning is particularly important in Sri Lanka where thousands of people die each year and preventing suicide has become a national public health priority [4,5]. The case fatality for self-poisoning in Sri Lanka is around 10%[1] but increases to over 50% for some pesticides [6]. More effective medical management is urgently required [7,8].

Current management of self-poisoning involves resuscitation and stabilisation of the patient, administration of antidotes where available, and gastric decontamination [9]. Mechanical forced emesis and gastric lavage are routinely used in Sri Lanka and other parts of Asia, despite little evidence for benefit [10,11]. Activated charcoal is available in some hospitals but is not routinely used in all, due to doubts about its effectiveness.

Animal and simulated human overdose studies have shown that a single dose of activated charcoal, if given soon after a poison is ingested, can reduce absorption of the poison [12]. The ability of charcoal to prevent absorption of poison falls off rapidly within one hour. Multi-dose regimens of charcoal may be effective much later for some poisons since the presence of activated charcoal in the intestine will interrupt the enterohepatic circulation [13,14] and may also draw poison out of the gut vasculature into the bowel [15].

At the time this RCT was designed, there had been no human studies of activated charcoal with clinically relevant outcomes. During 1997 and 1999, the American Academy of Clinical Toxicology and European Association of Poisons Centres and Clinical Toxicologists published reviews assessing the value of both single and multiple dose regimens of activated charcoal in acute poisoning [12,14]. Each position statement noted that they had been unable to find high quality studies with which to assess the clinical benefit of activated charcoal. The reports stressed the importance of establishing high quality RCTs with clinically relevant outcomes in order to determine the role of these interventions in poisoning management. The evidence for clinical benefit from a single dose of activated charcoal was again reviewed in 2004, but no new trials were reported [16].

We further carried out a *Clinical Evidence *search and appraisals in 2001 (and annually thereafter), together with systematic review of Medline, Embase, and Cochrane Collaboration databases, plus discussion with experts in the field, to identify relevant RCTs looking at activated charcoal in acute self-poisonings before starting our RCT [17]. We were unable to find any studies that were not discussed in the above reviews.

After this RCT started, two controlled studies of a single dose of activated charcoal in poisoning with pharmaceuticals were reported from the USA[18] and Australia [19]. Both used a primary outcome of length of hospital stay rather than a clinical outcome. The former study recruited 1479 patients but did not randomize them, instead using an 'even-odd day' system for allocation. A problem with this method is shown by the very different numbers of patients allocated to each arm, drawing doubt on the conclusion that activated charcoal had no effect. The latter study randomised 327 patients and found no difference between patients receiving charcoal and those receiving none. Neither study was relevant to the majority of patients seen in Sri Lanka.

15 months into the trial, a 400 patient RCT was published that reported benefit from 12 doses of activated charcoal in yellow oleander poisoning [20], a poison ingested by around 30% of patients in our RCT. After review of the published RCT and the results of an interim analysis of 595 oleander poisoned patients receiving either SDAC or MDAC in this trial, the IDMEC decided that it was important to obtain independent verification of the first study's result, and recommended that the trial continue.

We set up this RCT in Sri Lanka in early 2002 and recruited the first patient on the 31^st ^March 2002. The study was stopped in October 2004 after the final interim analysis with more than 4500 patients recruited. The final data analysis is underway and should be reported during 2007.

## Methods/design

The study was designed as an open RCT with three parallel groups: multiple dose charcoal vs single dose charcoal vs no charcoal in Sri Lankan adult patients presenting to a secondary hospital with a history of acute self-poisoning. From May 2004, an RCT of pralidoxime in symptomatic organophosphorus pesticide poisoned patients (ISRCTN55264358) was nested into this study.

The principal research question to be addressed was whether activated charcoal, either as a single or multi-dose regimen, will reduce the rate of death and complications following acute poisoning.

The trials were drawn up using the MRC's clinical trial proforma and were designed to be compliant with the CONSORT statement [21].

### Patients

The RCT was performed in Anuradhapura and Polonnaruwa General Hospitals, the secondary referral hospitals in North Central Province, and in Kurunegala Teaching Hospital, the secondary referral hospital for much of the North Western province and a tertiary referral hospital for areas further north.

All patients with a history of poison ingestion were approached concerning the study. Written informed consent was requested from conscious patients by a study physician in the patient's own language. For patients between 14 and 16 years, written informed consent was obtained from the patient's parents/guardian. As requested by the Faculty of Medicine Ethics Committee (see below), consent for unconscious adult patients was sought from accompanying relatives. Patients under the age of 16 or unconscious on admission and without any relatives were not recruited to the trial.

Patients who do not give consent to recruitment received usual care from the medical ward staff. Their progress was monitored at each ward round but the study team was not involved in their care.

### Inclusion and exclusion criteria

The study aimed to recruit all patients admitted to the adult medical wards of the study hospitals with a history of acute oral self-poisoning. Exclusion criteria were: age under 14 yrs (lower age of patients admitted to Sri Lankan adult medical wards); prior treatment with activated charcoal during this episode of poisoning; known pregnancy; ingestion of a corrosive; ingestion of a hydrocarbon alone; requirement for oral medication; inability of the medical staff to intubate the patient with Glasgow Coma Score (GCS) <13; presentation >72 hrs post-ingestion, and previous recruitment to the RCT. The last four exclusion criteria were added to the protocol in an amendment submitted to the ethics committee in May 2002.

### Patient management

All patients are seen on admission to the medical wards or, in Kurunegala, after admission to the Emergency Treatment Unit (emergency department). There the patients were resuscitated as necessary by wards doctors together with study doctors. The patient's airway was stabilised and oxygen, atropine, fluids, and antidotes given as necessary.

Gastric decontamination was only started when patients were stable. The original protocol stated that patients would receive neither gastric lavage nor forced emesis. However, at the request of the consultant physicians caring for the patients, from June 2003 patients who presented within two hours of ingestion of a potentially serious poison received a brief gastric lavage (3 × 300 ml of water). Many patients received either gastric lavage or mechanical forced emesis in the peripheral hospital transferring the patient before admission to the study hospitals.

Patients remained under the care of the hospitals' consultant physicians using management protocols agreed between the consultants and study team [22]. The ward medical teams made decisions about intubation and transfer of patients to intensive care or for cardiac pacing independently of study doctors. All decisions were made on the basis of clinical condition and do not reflect the poison ingested or the charcoal allocation.

Patients were seen regularly by full time study doctors at least every three hours and more often according to clinical need, 24 hrs a day. Patients were also seen on a study ward round twice each day (0830, 2030) at which time their condition was recorded in a handheld computer using a specially written database. Significant events (intubation, seizures, death) were recorded at the time of the event. The patients' condition over the previous 12 hrs was reviewed at each ward round.

Patients were first managed on the medical ward. Seriously ill patients, as judged by the medical team, were then transferred to the intensive care unit (ICU) as a bed became available. Each hospital had 2–8 ICU beds for medical patients; many were filled with OP poisoned patients and there was usually a delay in obtaining a bed.

Criteria for intubation included tidal volume less than 180 ml/breath using a Wright's respirometer, respiratory rate of less than 10 breaths/minute, abdominal breathing, or failure of non-invasive methods to maintain a patent airway. Arterial blood gas measurements were not available to guide therapy. Hypotensive patients, unresponsive to atropine and fluid resuscitation, were treated with dopamine plus dobutamine as necessary (these were the only inotropes available in the hospitals).

### Trial interventions and study procedures

Patients randomised to single or multiple dose activated charcoal regimens ingested 50 g of superactivated charcoal (Carbomix BP, Norit, NL; 2000 m^2^/g) in 300 ml of water (without sorbitol or mannitol) soon after recruitment. Patients receiving multiple doses subsequently ingested another five doses of 50 g charcoal at four hour intervals. The original RCT protocol proposed administering 18 doses of charcoal every 4 hours over 3 days. Early experience in the study demonstrated the immense difficulty of giving so many doses to patients (in particular gaining the patient's acceptance) and the number of doses was reduced to six in an amendment sent to the ethics committees in May 2002.

Alert patients drank the charcoal; patients with reduced consciousness received the charcoal via a nasogastric tube. Control patients did not receive activated charcoal. All patients were kept well hydrated to reduce the risk of intestinal blockage with the charcoal and monitored regularly throughout their time on the medical ward. Patients were followed up until final hospital discharge or transfer to a psychiatric ward.

We did not expect a major problem with compliance since the charcoal was given by the study team while the patient was under supervision in hospital. Unconscious patients received the charcoal by nasogastric tube after intubation. Conscious patients were actively encouraged to drink the charcoal by the study team; however, patients were not forced in any way to take the charcoal.

A 10 ml blood sample was taken using a sterile syringe and needle from each patient on recruitment. Further 5 ml blood samples were taken at one, four and twelve hours post-treatment, and then at daily intervals until discharge or death. Whether a needle or indwelling cannula was used was determined by the wishes of the patient. The identity and blood concentration of the poison was assayed retrospectively in only a subset of patients due to limited laboratory facilities.

### Randomisation

**Figure 1:**
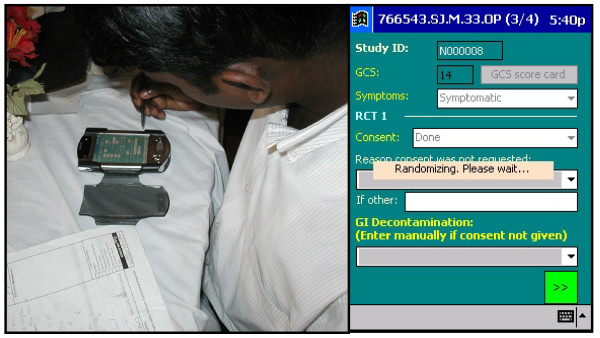
The hand held computer used to recruit and randomise patients and to record all events. Randomisation was performed at the patient's bedside. Allocation could not be predicted by the study doctor.

Patients were randomised into one of three study arms. The random allocation sequence was generated by computer and incorporated into a Microsoft Access programme written for patient recruitment, randomisation and event recording (Figure 1). Stratified block randomisation was performed using the following strata: (i) the toxin stated to have been ingested (oleander, organophosphate or carbamate, organochlorine, other pesticide or unknown pesticide or paraquat, medicine or unknown) ; (ii) reported time between poisoning and recruitment (<1 hr; 1–4 hrs; >4 hrs; unknown); and (iii) status on admission (Asymptomatic, Symptomatic with GCS 14 or 15/15, Symptomatic with GCS <14).

The allocation sequences were generated independently by the study statistician (EJ) and implemented by the programmer (SA), neither of whom had a role in patient recruitment, treatment or assessment. Variable block sizes of 3, 6 and 9 were used to allocate patients in equal numbers to each treatment group i.e. ratio 1:1:1 using Stata v7 software (ralloc subroutine version 3.2.5).

Participants were recruited and randomised by a study doctor at the bedside using a dedicated handheld computer at each study hospital (Figure 1). Randomisation occurred after the patient's baseline data had been entered and receipt of consent noted, and could not be manipulated by study doctors. The recruiting doctor was unable to predict allocation before randomisation.

All practical steps were taken to avoid bias: (i) the randomisation program was designed to be rapid and simple to operate, and yet remain independent of the investigators; (ii) the next treatment allocation could not be predicted in advance; (iii) the primary outcome, vital status at discharge, was unambiguous, and the secondary outcomes were objective; (iv) all outcomes were recorded systematically by the study team, not other hospital physicians; (v) patient follow-up was expected to be near 100% complete; and (vi) the analysis will be performed on an intention-to-treat basis.

### Outcomes

The primary outcome was all-cause mortality during hospital admission.

Secondary outcomes were identified *a priori *as relevant to specific poisons since different complications were expected according to the poison ingested. For pesticides, secondary outcomes included: (i) proportion of patients requiring intubation; (ii) total period of time ventilated; (iii) time to first ventilation; and (iv) seizures. For yellow oleander poisoning, the secondary outcome was the proportion of patients with cardiac dysrhythmias requiring anti-digoxin Fab (indications: 3° heart block, Mobitz type II 2° block, sinus bradycardia with heart rate <35 bpm, and sinus arrest or block with sinus pauses >3 secs) or serum potassium greater than 6.0 [23]. Unfortunately, anti-digoxin Fab became unavailable after four months [24] and patients were then transferred to tertiary cardiac centres for temporary pacing. Transfer used the same criteria and therefore this secondary outcome become a combination of receiving anti-digoxin Fab or transfer for tertiary care.

All secondary outcomes were reviewed twice a day at the review ward rounds. Any events not recorded at the time they occurred were recorded at this ward round.

### Sample size

In both hospitals, at least 10% of poisoned patients die before discharge [1]. An absolute reduction of 3% will be clinically important. In order to be able to detect whether either regimen of activated charcoal reduced the death rate from 10% to 7%, with a significance level (alpha) of 5% and a power of 80%, a minimum of 1400 patients had to be recruited to each arm of the trial (i.e. 4,200 patients in total).

Sample size calculations were based on current practice and a case fatality rate of 10%. The Independent Data Monitoring and Ethics Committee (IDMEC) was asked to review the recruitment and event rates during the first year of the trial in order to make recommendations about expanding the trial by including further hospitals if necessary.

There should have been no loss to follow up for the primary outcome. Patients were either discharged alive from the wards or their bodies transferred to the morgue for judicial autopsy. All secondary outcomes were assessed in hospital before discharge.

### Study hypotheses and principal comparisons

The main hypothesis is that multi-dose activated charcoal will reduce the case fatality rate from 10% to 7%, hence the first principal comparison will be multi-dose activated charcoal versus no intervention. The potential of multidose regimens to work long after ingestion – due to interruption of enterohepatic circulation and gut dialysis – means that such a regimen is more likely to work in a situation where people typically present several hours after ingestion.

We suspected that a single dose of charcoal would be less effective since most poison absorption will have taken place by the time the patients present to hospital. Therefore, the second principal comparison will test the hypothesis that the case fatality rate in patients receiving a single dose of activated charcoal is equal to that in patients receiving multiple doses.

In order to investigate whether a single dose of activated charcoal has a similar effect as giving no intervention, the third principal comparison will test the hypothesis that the case fatality rate in patients receiving a single dose of activated charcoal is equal to that in patients receiving no intervention.

### Statistical analysis

The main analysis will be carried out on an intention-to-treat basis, using the chi -squared test for the primary outcome (or Fisher's exact test if appropriate) and for other dichotomous outcomes. Odds ratios (plus 95% confidence intervals) will be reported to establish magnitude and direction of the treatment effect. In the original protocol, we indicated that risk would be the chosen measure of effect; however, in order to be able to adjust the treatment effect estimate for stratification variables in future analyses, we will calculate odds instead, using logistic regression. Since the primary outcome is an uncommon event, risk and odds should be similar.

For outcomes where time-to-event is recorded, we will use the logrank test to compare treatment groups, produce Kaplan-Meier curves to illustrate the comparison, calculate incidence rates and perform Cox's regression to estimate hazard ratios, adjusted for stratification factors.

An analysis of trends in treatment effect for factors 'reported time from ingestion to treatment' and 'patient status on admission' will be performed.

It is possible that both treatment regimens, if effective in reducing case fatality rates, will be more effective the earlier they are started. Therefore we will assess the trends in clinical effectiveness according to time post-ingestion to start of therapy using statistical modelling techniques. In order to determine whether treatment should be started irrespective of severity, we will likewise also assess trends in case fatality rates across a gradient of severity.

Admission blood samples will be retrospectively analysed to determine the identity of the poison ingested. The primary analyses will be repeated using the confirmed identity of the poison.

### Pre-specified subgroup analysis

Subgroup analyses are planned to look at the consistency of treatment effect across different types of poison suspected/reported to be ingested, time since ingestion and severity. Poison subgroups investigated will include organophosphorus pesticides, oleander, other or unknown pesticide' and paraquat, and other/medicine poisonings. A further subgroup of organophosphorus poisonings, diethyl versus dimethyl versus unknown will also be investigated. These subgroup analyses will use the test of interaction (or test for trend) and will be carried out for the primary outcome only.

### Independent Data Monitoring and Ethics Committee (IDMEC)

An independent IDMEC was established for the trial. For the duration of recruitment, interim analyses were supplied by the trial statistician (EJ), in strict confidence, to the Chair of the IDMEC, together with any other analyses the IDMEC requested. Meetings were arranged periodically, as considered appropriate by the Chair. In the light of interim data, and other evidence from relevant studies, the IDMEC was to inform the principal investigator (Dr Michael Eddleston), if in their view (i) there was proof beyond reasonable doubt that the data indicated that any part of the protocol under investigation was clearly indicated or contra-indicated, either for all participants or for a particular subgroup of trial participants, or (ii) it was evident that no clear outcome would be obtained.

The decision to inform the principal investigator in either of these circumstances was, in part, based on statistical considerations. Appropriate criteria for proof beyond reasonable doubt were not specified precisely. A difference of at least three standard deviations in the interim analysis of the major endpoint might be needed to justify halting, or modifying, such a study prematurely. If this criterion were to be adopted, it would have the practical advantage that the exact number of interim analyses would be of little importance, and so no fixed schedule is proposed [25]. Unless modification or cessation of the protocol was recommended by the IDMEC, the principal investigator, co-investigators, collaborators and administrative staff were to remain ignorant of the results of the interim analyses. Collaborators and all others associated with the study could write to the Chair of the IDMEC to draw attention to any concern they may have about the possibility of harm arising from the treatment under study, or any other matter that may be relevant. The principle investigator would follow the advice of the Chairman concerning decisions to continue or stop the trial.

The members of the IDMEC for this study were:

Dr Mike Clarke, Cochrane Collaboration, Oxford, UK (Chairman).

Dr Julian Higgins, MRC Biostatistics Unit, Cambridge, UK (Statistician).

Professor Keith Hawton, Dept Psychiatry, Oxford University, UK.

Professor Saroj Jayasinghe, Dept Medicine, Colombo University, Sri Lanka.

Professor Nimal Senanayake, Dept Medicine, Peradeniya University, Sri Lanka.

Professor Krisantha Weerasuriya, SEARO/WHO, Delhi, India.

### Ethics

Ethics approval was received from the Oxfordshire Clinical Research Ethics Committee, UK (application number C01.120), and from the Faculty of Medicine Ethics Committee, University of Colombo, Sri Lanka (number EC_00_77)

## Discussion

If activated charcoal can be proven to be effective, then it should be an extremely valuable therapy since it is widely available in the developing world, relatively cheap, binds to many poisons, and safe once the airway is protected. Since the greatest benefit has been seen when charcoal is given within 15 mins of poisoning,[12] it could be supplied to villages to allow people to give charcoal to their relatives within minutes of the poisoning and before transfer to hospital. On the other hand, if it is found to be ineffective, then the many thousands of dollars currently being spent on activated charcoal across Asia could be diverted to more effective interventions.

One major issue with this trial was that neither gastric lavage nor mechanical forced emesis was to be given to trial patients, following current international practice. Recent systematic reviews by international toxicological societies [10,11] (updated 2004 [26,27]) were unable to find any evidence that either gastric lavage or forced emesis with ipecacuanha produces clinical benefit.

Human simulated overdose studies indicate that around 30% of tablets can be washed out if gastric lavage is initiated within 15 mins of poisoning. However, the yield falls off rapidly after this time [11,26]. The yield from liquid poisons such as pesticides is likely to be even less since liquids pass out of the stomach into the small bowel (and therefore out of the reach of lavage) quicker than the tablets on which most studies have been performed. This is likely to be particularly true for OP pesticides since their cholinergic action speeds up gastric emptying.

Serious complications such as oesophageal rupture and aspiration occur with gastric lavage, particularly when the procedure is performed in uncooperative conscious patients or in unintubated unconscious patients [11,26]. Insertion of gastric lavage tubes also risks generating a bradycardic vasovagal response, sometimes resulting in asystole. This can be particularly severe in patients with OP or oleander poisoning who already have poison-induced bradycardias. An observational series of gastric lavage in 15 Sri Lankan patients revealed 2 deaths due to the lavage and nine cases of aspiration requiring antibiotics [28]. The risks of gastric lavage in circumstances when resources limit the ability of doctors to sedate and intubate patients far outweigh the small benefits possible from the procedure.

The use of ipecacuanha for forced emesis has been discouraged due to complications of persistent vomiting in patients at risk of rapid reductions in conscious level and its poor return. The yield of forced emesis without ipecacuanha is minimal and for these reasons forced emesis of any form is now discouraged [10,27].

After discussion, this approach was approved by both ethics committees. However, some clinicians considered lavage to be of fundamental importance to medical management and practice was changed after 15 months to follow that recommended in a Sri Lankan textbook of poisoning. Thereafter, all patients who presented within two hours of a serious ingestion underwent gastric lavage after stabilisation.

## Competing interests

The author(s) declare that they have no competing interests.

## Authors' contributions

ME designed and established the poisoning cohort in Sri Lanka, designed this study, and wrote the grant application. EJ helped design the RCT, conducted interim analyses and will perform the final statistical analysis, producing a statistical report. NAB helped design and establish the cohort study and RCT. FM and LS ran the trial centres. SA wrote the Access program for recruitment, randomisation, and data collection. WD, AH, SA, KJ, and SJ were consultant physicians in the study hospitals and had clinical responsibility for the patients. MHRS and DAW are the senior members of the Ox-Col collaboration. All authors had a role in improving the study design.

## Pre-publication history

The pre-publication history for this paper can be accessed here:


